# Cdk4/6 inhibitors and overall survival: power of first-line trials in metastatic breast cancer

**DOI:** 10.1038/s41523-018-0068-4

**Published:** 2018-06-26

**Authors:** Marie-Laure Tanguy, Luc Cabel, Fréderique Berger, Jean-Yves Pierga, Alexia Savignoni, Francois-Clement Bidard

**Affiliations:** 1 Department of Biometry, Institut Curie, PSL Research University, Saint Cloud, France; 2 Department of Medical Oncology, Institut Curie, PSL Research University, Paris, France; 3 UVSQ, Paris Saclay University, Saint Quentin en Yvelines, Paris, France; 4 Paris Descartes University, Paris, France

## Abstract

Palbociclib, ribociclib, and abemaciclib have been investigated in combination with aromatase inhibitors as first-line therapy for metastatic hormone receptor-positive breast cancer (PALOMA-2, MONALEESA-2 and MONALEESA-7, MONARCH-3 trials, respectively); pivotal trials led to absolute median progression-free survival (PFS) gain of about 15 months. We aimed to estimate, for each trial, the statistical power to demonstrate a significant gain in overall survival (OS). Power was calculated with Freedman’s formula. Given the allocation ratio and the number of events, power was computed as a function of hazard ratio. We focused on four specific hazard ratio values (0.94, 0.89, 0.81, and 0.77), which are estimated to correspond to absolute 3, 6, 12, and 15 months gain in OS, respectively. For these calculations, the type I error rate was stated at 5% with a two-sided test, and we assumed that the risk of death was constant over time. PALOMA-2 and MONALEESA trials have an almost similar power despite different allocation ratios, while MONARCH-3 has a more limited power. Overall, the power of the four trials to demonstrate a statistically significant improvement in OS is less than 70% if the prolongation in median OS is ≤12 months, whatever the OS data maturity. This analysis shows that OS results are jeopardized by limited powers, and a meta-analysis might be required to demonstrate OS benefit. Conversely, if a significant OS improvement is observed in some but not at all trials, this discrepancy might be more attributable to chance than to a truly different drug efficacy.

## Introduction

Endocrine therapies are the cornerstone of hormone receptor-positive (HR+) HER2-negative (HER2−) breast cancer treatment at both early and metastatic stages. Endocrine therapies for metastatic breast cancer (MBC) have remained largely unchanged for the past two decades, and include tamoxifen, aromatase inhibitors (AI), and fulvestrant.^1^ In 2012, results of BOLERO-2, a randomized placebo-controlled phase 3 conducted in patients with HR+ HER2− MBC progressing under first-line nonsteroidal AI, have been reported.^2^ This trial compared the efficacy of a steroidal AI (exemestane) to that of a combination of exemestane and everolimus, a mTOR inhibitor. Patients in the everolimus-exemestane arm had a significantly longer PFS, with a hazard ratio (HR) = 0.43, 95% CI [0.35; 0.54].^2^ In that second-line setting, despite a 4.6-month prolongation in median PFS, adding everolimus to exemestane did not confer a statistically significant improvement in the overall survival (OS): HR = 0.89, 95% CI [0.73; 1.10].^3^ This negative result increased the concerns about the limited cost-effectiveness of everolimus in that setting.^4,5^

More recently, further significant progresses have been reported in HR+ HER2− MBC: four randomized phase 3 trials have reported superior progression-free survivals (PFS) for AI and cdk4/6 inhibitors combinations compared to AI and placebo as first-line therapy. The PALOMA-2 trial, in which 666 patients have been randomized 2:1 between the AI and palbociclib arm and the AI and placebo arm, was the first to be reported and demonstrated a PFS HR of 0.58, 95% CI [0.46; 0.72].^6^ In the MONALEESA-2 trial, 668 patients have been randomized in a 1:1 fashion between the AI and ribociclib arm and the AI and placebo arm, with a PFS HR of 0.56, 95% CI [0.43; 0.72].^7^ Superimposable number of included patients and results have been reported with ribociclib in a second pivotal trial, MONALEESA-7, that was conducted in premenopausal women [8]. Recently, in the MONARCH-3 trial, 493 patients have been randomized in a 2:1 fashion between the AI and abemaciclib arm and the AI and placebo arm, with a PFS HR of 0.54, 95% CI [0.41;0.72]^8^ (Table 1). Based on these significant PFS improvements, cdk4/6 inhibitors have been approved by regulatory agencies for first-line HR+ HER2− MBC and are now being largely used in that setting. However, in the context of a metastatic disease, and not withstanding quality of life-related endpoints, the ultimate goal of a palliative therapy is to extend OS, while PFS is moderately correlated with OS.^9^ In all three trials, OS was defined as a secondary endpoint, and no mature data have been reported so far (20% of deaths were observed in the last MONALEESA-2).^10^

**Table 1:** Summary of trial characteristics and results

	PALOMA-2 trial	MONALEESA-2 trial	MONALEESA-7 trial	MONARCH-3 trial
cdk4/6inh	Palbociclib	Ribociclib	Ribociclib	Abemacicl
Number of patients AI + placebo arm	222	334	337	165
Median PFS AI + placebo arm	14.5 months	14.7 months	13 months	14.7 months
Number of patients AI + cdk4/6inh arm	444	334	337	328
Median PFS AI + cdk4/6inh arm	24.8 months	Not reached	23.8 months	Not reached
PFS hazard ratio [95%CI]	0.58 [0.46; 0.72]	0.56 [0.43; 0.72]	0.55^a^	0.54 [0.41; 0.72]

*cdk4/6in*h cdk4/6 inhibitor, *AI* aromatase inhibitor

^a^Confidence interval not available at time of analysis

Per protocol, 278 OS events (41% maturity) and 315 OS events (47% maturity) will trigger the main OS analysis in PALOMA-2 and MONALEESA-2, respectively. In the MONARCH-3 trial, the main OS analysis is planned as part of a pooled analysis with the MONARCH-2 study; OS analyses of MONARCH-3 as a single study could be reported as exploratory analyses with no prespecified maturity. As for many trials and despite methodological concerns, it is very likely that unplanned OS analyses will be reported even after the main analyses have occurred.

In this report, we estimated the power of each of the three trials to demonstrate a significant gain in OS according to their intrinsic design (number of patients included, randomization ratio), and the number of events.

## Results

**Fig. 1:**
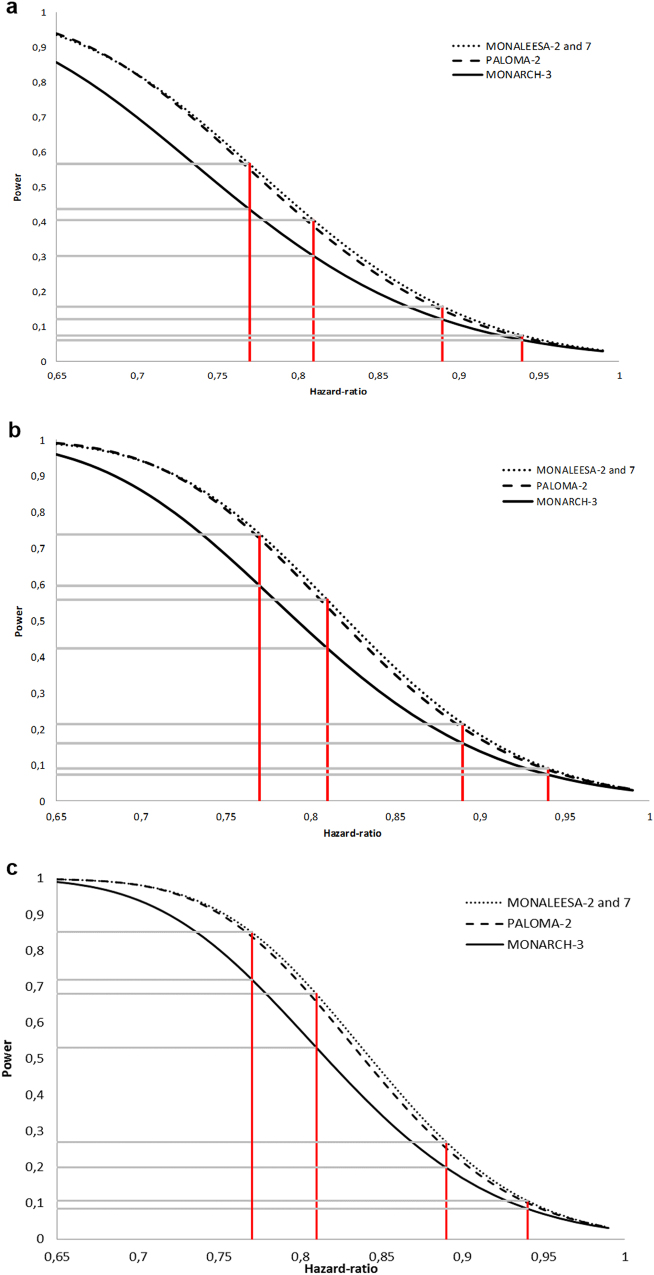
Power to demonstrate a benefit on overall survival. **a**–**c** Power at 40, 60, and 80% death rate (maturity), respectively. HR of 0.94, 0.89, 0.81, 0.77, which are estimated to correspond to absolute 3, 6, 12, and 15 months gain in OS (on the basis of a 50 months median OS in control arms^12^) are highlighted in red

Figure 1 depict, for each OS data maturity and for each of the three phase 3 trial, the power to demonstrate a statistically significant improvement in OS according to different HRs. As expected, the higher the OS maturity, the higher the power is for a given HR. Unsurprisingly, MONALEESA-2 and -7, which share similar design and number of treated patients, have superimposable powers. We observed that PALOMA-2 and MONALEESA trials have an almost similar power, MONALEESA trials displaying a marginally increased power. We confirm that the MONARCH-3 trial is less powerful than the three other trials.

For each of the four specific HR values proposed, the power of each trial is displayed on Table 2. Whatever the OS data maturity, if the prolongation in median OS is less than 12 months, the four trials have a power less than 70%. For a prolongation of 15 months, a power of 80% or more is reached for a death rate of 80%. The power of MONARCH-3 is about 72%.

**Table 2:** Examples of power estimates

Hazard ratios	**Death rate**	PALOMA-2 trial	MONALEESA-2 trial	MONALEESA-7 trial	MONARCH-3 trial
**0.77**	40%	0.55	0.57	0.57	0.44
(15 months gain in median OS)	60%	0.73	0.74	0.74	0.60
	80%	0.84	0.85	0.85	0.72
**0.81**	40%	0.39	0.40	0.40	0.30
(12 months gain in median OS)	60%	0.54	0.56	0.56	0.42
	80%	0.66	0.68	0.68	0.53
**0.89**	40%	0.15	0.16	0.16	0.12
(6 months gain in median OS)	60%	0.20	0.21	0.21	0.16
	80%	0.25	0.27	0.27	0.20
**0.94**	40%	0.07	0.07	0.07	0.06
(3 months gain in median OS)	60%	0.09	0.09	0.09	0.07
	80%	0.10	0.11	0.11	0.08

Calculations were made on the basis of a 50 months median OS in control arms [12]. The power values displayed in this table are also displayed Fig. 1

## Discussion

In the absence of cure, the ultimate goals of metastatic cancer therapy are to improve duration and/ or quality of survival of the patients. New anticancer drugs should therefore demonstrate a benefit on OS and/or an improvement of the quality of life. Other clinical endpoints such as PFS may be useful intermediates, but are not surrogates of OS and, in MBC, the level of evidence supporting a surrogacy of any biomarker,^9^ including circulating tumor cells count is low.^11^

Here we showed that MONARCH-3 is less powerful than PALOMA-2, MONALEESA-2, and MONALEESA-7 trials, and that these three latter trials have an almost similar power; the small difference in power between them is explained by the difference in treatment allocation ratio: 2:1 in the PALOMA-2 trial vs 1:1 in the MONALEESA trials, the three trials having enrolled a similar number of patients.

PALOMA-2 and MONALEESA trials have a <0.70 power to report a statistical difference if the observed absolute PFS gain (about 15 months, in first line) is translated into a 12-month OS improvement; smaller OS improvements will be more difficult to demonstrate. A power of 80% is observed only if the OS improvement is 15 months or more. Depending on the final OS HR achieved by cdk4/6 inhibitors, the more limited power of MONARCH-3 may be responsible for a scenario in which a statistically significant OS gain is observed in PALOMA-2 and MONALEESA trials, but not in MONARCH-3—even if there is no true efficacy difference among the three cdk4/6 inhibitors. Our analysis illustrate that, OS being the preferred outcome, it will not be satisfactory when median OS is long and differences need to be very large to demonstrate an effect. Such comparisons are further complicated by the numerous post-progression therapies available in MBC, which will make the two arms more similar. A gain in OS is much more likely to be demonstrated by pooling individual patient data from different trials. A pooled analysis of MONALEESA-2 and MONALEESA-7 trials will be probably be reported in a near future, as both trials tested the same drug. A larger meta-analysis including the individual data from all patients including the four trials would have a very high statistical power to demonstrate that cdk4/6 inhibitor do increase OS. If not made mandatory by regulatory agencies, the feasability of such meta-analysis will rely on the willingness of three competing pharmaceutical companies.to collaborate.

Our analyses have several limitations, as many parameters (post-first line therapies, treatment with cdk4/6 inhibitors at a later stage…) will ultimately influence the OS differences between the two arms of each trial and between trials. A key parameter will be the OS reached by control arms, which is currently unknown. The PALOMA-2 study hypothesized that a median OS of 34 months in the control arm of this international trial, while our analyses were based on real life OS data obtained in France in that specific population of AI-sensitive metastatic MBC patients (50 months).

In conclusion, power to demonstrate a gain in OS limited in these trials; ultimately, if a significant OS improvement is observed in some but not at all trials, this discrepancy might be more attributable to chance than to a truly different drug impact on OS.

## Methods

Power was calculated with Freedman’s formula, which allows us to compute power for studies comparing survivor functions of two groups by using the log-rank test. Given the allocation ratio and the number of events, power was computed as a function of HR. For these calculations, the type I error rate was stated at 5% with a two-sided test, and we assumed that the risk of death was constant over time. We particularly focused on four hypothetic prolongations in median OS following the use of cdk4/6 inhibitors: 3, 6, 12, and 15 months. To translate HR into clinically meaningful data, we hypothesized that the median OS in control arms will be equal to 50 months, as observed in more than 6000 AI-sensitive HR+ HER2− MBC patients treated prior to the cdk4/6 inhibitor era.^12^ According to the prolongations proposed above, the four HR of interest are 0.94, 0.89, 0.81, and 0.77, respectively. We calculated the power for two hypotheses of death rates occurring over the follow-up period: 40, 60, and 80%, reflecting the OS data maturity. The analysis was performed using R software version 3.3.2 [13].

### Data availability statement

All data generated or analyzed during this study are included in this published article.
